# Detection of human papillomavirus genes in human oral tissue biopsies and cultures by polymerase chain reaction.

**DOI:** 10.1038/bjc.1989.146

**Published:** 1989-05

**Authors:** N. J. Maitland, T. Bromidge, M. F. Cox, I. J. Crane, S. S. Prime, C. Scully

**Affiliations:** Department of Pathology, Medical School, Bristol, UK.

## Abstract

**Images:**


Br. J. Cancer (1989), 59, 698–703 © The Macmillan Press Ltd., 1989

### Detection of human papillomavirus genes in human oral tissue biopsies and cultures by polymerase chain reaction

N.J. Maitland[1], T. Bromidge[1], M.F. Cox[1,2], I.J. Crane[2], S.S. Prime[2] & C. Scully[2]

[1]*Department of Pathology and Unit of Molecular Genetics, The Medical School and* [2]*University Departments of Oral Medicine, Surgery and Pathology, University of Bristol, Bristol BS8 1TD, UK.*

**Summary** We have used the polymerase chain reaction to detect DNA sequences related to human papillomavirus type 16, by simultaneous priming with oligonucleotides from the E6 and L1/L2 open reading frames of the HPV16 genome. The HPV16-related sequence is present at low levels in normal oral tissue, in addition to biopsies and cell cultures from patients with benign and malignant disease. Ultimate analysis of the amplified sequences from the E6(120bp) and L1/L2(173bp) regions of HPV16 was achieved by gel electrophoresis and comparative nucleotide sequencing. The oral carcinoma biopsies and tissue cultures contained DNA sequences which were identical to the E6 region of HPV16, but only rarely contained sequences closely related to the L1/L2 region. The PCR technology should permit the detection, identification and cloning of latent viruses from extremely small tissue biopsies.

Epidemiological studies have implied that an infectious agent may play a role in human oral cancer (reviewed by Scully *et al.*, 1988). A similar association has been noted between human cervical cancer and a specific infectious agent, namely human papillomavirus (HPV), in particular HPV type 16 (Howley, 1986). In the United Kingdom, up to 80% of invasive cervical carcinomas probably contain HPV16 DNA (reviewed in Munoz *et al.*, 1988) hence the implication of HPV16 as an aetiological agent in the disease. In oral lesions, HPV antigens and genes have been detected, principally in benign papillary lesions (Loning *et al.*, 1985), but also in carcinoma tissue (de Villiers *et al.*, 1985). Our own studies (Scully *et al.*, 1987; Maitland *et al.*, 1987*a*) indicated that a HPV, closely related to HPV16, was present as DNA at low levels in oral tissue types, ranging from histologically normal through benign leukoplakia to invasive carcinoma. If an aetiological role for HPV is proposed in oral cancer, it is necessary to determine the incidence of the virus in the affected population and to follow the interactions between HPV and the oral epithelium. Detailed epidemiological study, in contrast to simple screening, requires either large amounts of material or more sensitive techniques than those presently available, and it was with these aims in mind that the present work was undertaken.

First, to overcome the shortage of material, we have established keratinocyte cultures from oral squamous cell carcinoma tissue according to the method described by Rheinwald and Green (1975) and modified by Crane *et al.* (1986 and manuscript in preparation). Secondly, we have developed the polymerase chain reaction (PCR), as originally described for the detection of point mutations in haemoglobinopathies and cellular oncogenes (Chehab *et al.*, 1987; Saiki *et al.*, 1986, 1988; Bos *et al.*, 1987). The PCR depends on the *in vitro* amplification of small segments of DNA, located between oligonucleotide primers of known DNA sequence. Using this technology we can detect a single copy of the HPV genome in $10^4$ uninfected cells, or in a biopsy of only 25 cells (Young *et al.*, unpublished manuscript). The use of PCR and oligonucleotide hybridisation to detect HPV DNA in histological sections of human cervical carcinoma has recetly been published (Shibata *et al.*, 1988). Using primers located in the E6 gene, which is preferentially retained and expressed in cervical carcinoma, and L1/L2 overlap region of HPV16 (Seedorf *et al.*, 1985; Smotkin & Wettstein, 1986) we provide further evidence, at the nucleotide base sequence level, that human oral tissues and tissue cultures contain the 'cervical' HPV type 16 DNA.

Correspondence: N.J. Maitland.
Received 19 August 1988, and in revised form, 28 November 1988.

### Materials and methods

### Source of tissues and cell cultures

Cubes of freshly frozen (in liquid $N_2$) tissue biopsies were obtained from the Bristol Dental Hospital (oral samples), Birmingham Maternity Hospital (cervical cancers) and Bristol Royal Infirmary (cervical smears). Duplicated independent histological analysis was obtained for all the oral and cervical cancer samples. The oral tumours reported here were all classified as well differentiated squamous cell carcinomas. In all cases the tissue extracted for DNA was adjacent to that from which frozen sections were cut for histology to minimise the effects of tissue heterogeneity on the extracted material.

Keratinocyte cultures were established from human oral squamous cell carcinoma tissue, and passaged without cloning as described by Crane *et al.* (1986) for rat oral squamous cell carcinoma tissue. DNA was extracted from healthy subconfluent cultures at various passages after initial establishment as a primary culture (see below). Cell cultures of CaSki were obtained from the ATCC.

### Extraction and purification of DNA

Nucleic acids (both DNA and RNA) were extracted according to a variation of the method of Chirgwin *et al.* (1979) as previously described (Maitland *et al.*, 1987*b*) from all of the tissue cultures and biopsies. Briefly, this involved homogenisation of the tissue in a detergent/guanidinium thiocyanate mixture, followed by ultracentrifugation for 16 h through a double cushion of caesium trifluoroacetate (Pharmacia) containing ethidium bromide. DNA formed a u.v. fluorescent band between the two cushions, whereas the RNA formed a solid pellet at the bottom of the centrifuge tube. This modification allows the maximum information to be obtained from a small (1 $mm^3$) tissue biopsy.

### Polymerase chain reaction

PCR was carried out essentially according to the manufacturer's instructions for 30 cycles of denaturation (30 s at 91°C), annealing (30 s at 25°C) and synthesis (5 min at 55°C) in a buffer containing 16.6 mM ammonium sulphate, 67 mM Tris.HCl pH 8.8, 6.7 mM $MgCl_2$, 10 mM mercaptoethanol, 6.7 $\mu$M ethylene diamine tetraacetic acid (EDTA), 33 $\mu$M each of dATP, dCTP, dGTP and dTTP, 10% dimethylsulphoxide (DMSO), 0.5–1.0 $\mu$g of human DNA target and 300 ng of each primer DNA (the reaction will work with considerably lower primer concentrations) in a final volume of 50 $\mu$l. After the reaction mixture is prepared, and the first denaturation (7 min at 91°C) completed, 1 unit of Taq DNA polymerase (Anglian Biotec) was added and the annealing, synthesis and denaturation were carried out for 10 cycles, when a second 1 unit of Taq polymerase was added. After 20 cycles a further 1 unit of the polymerase was added and the cycles continued up to 30. After this time half of the reaction products were removed, 1/10 volume of gel loading dye (0.25% bromophenol blue, 0.25% xylene cyanole, 25% Ficoll 400) was added to each and the samples were analysed on a polyacrylamide gel (12%) prepared in Tris/borate buffer (89 mM Tris-borate, 89 mM boric acid, pH 7.8) by electrophoresis at 45 mA for 60 min. The final amplified oligonucleotide products were visualised by ethidium bromide stain (1 $\mu g ml^{-1}$ in Tris/borate buffer) and ultraviolet illumination.

*Nucleotide sequence analysis of PCR products*

The 298 bp amplification product from the E6 gene primers 2 and 3 was purified from a 12% polyacrylamide gel by excision of the band, overnight elution in an excess of 0.1 × TE buffer at 37°C and centrifuge driven dialysis on a centricon 30 (Amicon) as described in Higuchi *et al.* (1988). The 40 $\mu l$ product of this was freeze dried, resuspended in up to 8 $\mu l$ of dd $H_2O$ and approximately half of this (depending on the yield) was transcribed *in vitro,* using T7 RNA polymerase (Davanaloo *et al.,* 1984). Sequencing of the *in vitro* transcripts was carried out with dideoxynucleotide nucleotide triphosphates and avian myeloblastosis virus reverse transcriptase (Pharmacia) exactly as described by Stoflet *et al.* (1988), using a $^{32}P$ end-labelled primer 2.

**Figure 1** Oligonucleotide primer sequences and locations on the HPV16 genome (adapted from Seedorf *et al.*, 1985) (**a**) E6 gene primers, (**b**) L1/L2 overlap region primers, (**c**) T7 polymerase sequencing primer position in the E6 gene.

### Results

### *Standard polymerase chain reaction on oral tissue DNA using HPV16 E6 gene primers*

One microgram of DNA from a number of different tissues and lesion types was subjected to the standard PCR at 55°C. We selected the E6 region of the HPV16 genome for amplification, and in particular a 120 bp fragment located close to the 3′ terminus as shown in Figure 1a. The expected 120 bp amplification product was visualised by ultraviolet illumination of the ethidium bromide stained polyacrylamide gel (Figure 2), except in the fetal kidney and the carcinoma culture 145(6). The negative result with 145(6) was in contrast to the positive result with 145(8). However, reamplification of the non-visualised products from 145(6) resulted in a weak positive result. Since 145(6) is an earlier passage of 145(8), it implies that selection of HPV16 positive cells may occur *in vitro*.

### *DNA sequence analysis of PCR products from the E6 region*

Preliminary comparative analysis of the PCR products from oral and cervical tissues was carried out with the restriction endonucleases shown in Figure 1a (data not shown). Since the amounts of DNA obtained from a single PCR were close to the limits required for convincing restriction endonuclease analysis, we were anxious to confirm the identity of the DNA amplified by the E6 primers as HPV16 at the ultimate level, i.e. that of the sequence of the nucleotide bases in this region. Initial experiments, in which the 120 bp E6 amplification products were directly sequenced with the sequenase system, produced disappointing results with material amplified from oral samples (whereas cervical samples gave correct sequences according to Seedorf *et al.* (1985) for both the E6 and L1/L2 genes). The lack of success with oral samples was attributed to the poor yields of PCR product and, to amplify these products further, we employed a third E6 primer, which was homologous to a 20 base sequence, 5′ to the original primer 1. Attached to the HPV16 portion of this primer was a 28 base sequence, encoding the T7 phage RNA polymerase promoter (Davanaloo *et al.*, 1984). After purification of the 298 bp amplification product of this reaction, it was therefore possible to amplify the products by *in vitro* transcription. Sequencing was accomplished with AMV reverse transcriptase from end-labelled primer 2 (Stoflet *et al.*, 1988). The results of a sequencing of one oral sample, and an HPV16 positive cervical carcinoma biopsy control are shown in Figure 3. Over the entire legible range, the two sequences were identical (the locations of primer 1 and the restriction endonuclease sites from Figure 1 are also indicated).

**Figure 2** Polymerase chain reaction with E6 primers 1 and 2. Reactions were performed on purified DNA from the sources indicated with the synthesis step carried out at 55°C. Analysis of the products was by 12% polyacrylamide gel electrophoresis. The position of molecular weight marker is shown arrowed on the left of the figure.

**Figure 3** Nucleotide sequence analysis of E6 amplification products from oral and cervical carcinoma DNA. Sequencing was carried out on the 298 bp product of a PCR with E6 primers 2 and 3 (see Figure 1c). The end-labelled sequencing primer was primer 2. The position of primer 1 in the determined sequence, and the restriction endonuclease cleavage sites from Figure 1a are also indicated.

### *Polymerase chain reaction on oral DNA samples using L1/L2 primers*

To test whether the late regions of the HPV16 sequence were present in the oral tissues, we employed primers synthesised from the overlap region between the L1 and L2 regions (Figure 1b). The results of the PCR with these primers are shown in Figure 4. Positive controls with CaSki DNA and cloned HPV16 DNA were positive for both the early and late primers separately and on simultaneous priming with all four oligonucleotides, to give the expected 120 and 173 bp amplification products respectively. Similar results were obtained with other cervical tumour DNA samples which contained considerably lower amounts (one to two genomes per cell) of HPV 16 DNA per cell (data not shown). In contrast, DNA from the oral tissues and cultures was positive only for the E6 diagnostic fragment, even under the less stringent reaction conditions, with the possible exception of culture H157(17) and SCC biopsy 1, which were weakly positive with the L1/L2 primers. In our experience, this implies an extremely low level of late genes, perhaps in a small number of the cells in the biopsy or culture, whereas early genes are present in the majority of the cells. In some of the oral samples, a number of low molecular weight bands (<80 bp) were detected. However, these bands failed to react with a HPV16 DNA probe (data not shown). Multiple low molecular weight bands of this nature were often observed, particularly when several sets of primers were employed simultaneously, but a positive reaction was only scored when the amplification product predicted from the published DNA sequence was observed.

**Figure 4** Simultaneous polymerase chain ractions using primers for E6 and L1/L2 regions on oral carcinoma culture and biopsy DNA. Analysis of the PCR products was carried out on a 12% polyacrylamide gel. Predicted sizes of the amplification products are shown arrowed on the left.

### Discussion

Our previous results (Scully *et al.*, 1987; Maitland *et al.*, 1987*a*) indicated that papillomavirus DNA, closely related to, but not identical to that from the potentially oncogenic cervical HPV type 16, could be detected in nucleic acid preparations from a number of oral lesions, including cancers. To further explore the nature of the oral variant of HPV16, we produced cultures from the tumour tissues, but even in these uncloned keratinocyte cultures, detection of HPV DNA was technically difficult. The weak signals could have been due to nucleotide base sequence differences between the cervical HPV16, which we used as a hybridisation probe, and the related oral virus. Equally, the positive results could have resulted from cross-hybridisation between human DNA sequences and the viral probe. By employing the PCR we were able to produce unequivocally positive results, in almost 50% of oral carcinomas tested, even with the smallest oral tissue biopsies. This value is entirely in agreement with our earlier results (Maitland *et al.*, 1987*a*).

An alternative explanation for the consistently poor signals obtained in experiments to detect HPV in oral tissues may lie in the distribution of viral DNA positive cells in the cultures and the biopsies. In some of the oral keratinocyte cultures the strength of the PCR signal for HPV16 E6 varied with the passage number of the cultures, implying selection for HPV-positive cells with time in culture. It is likely that an uneven distribution of HPV positive cells does occur, both *in vivo* and *in vitro,* based on this result and also on *in situ* hybridisations to HPV16 positive tissue sections (unpublished observations).

The design of oligonucleotide primers for the PCR technique used in this study was based on our previous results (Maitland *et al.*, 1987*a*). The E6 gene sequences are consistently retained in both cervical and oral carcinomas (Baker *et al.*, 1987; Smotkin & Wettstein, 1986; N. Maitland, unpublished data), whereas the late genes (L1/L2) are often deleted in cervical carcinomas when the HPV16 genome is integrated into the cell genome. In addition, only a very small proportion of the oral samples in our previous study contained detectable HPV16 late genes.

Using this combination of oligonucleotide primers the results again bore out those of our previous work, in that early genes were readily detected, whereas the late genes were freqently absent in oral tissues. Confirmation of the identity of the viral DNA present in the oral tissues was achieved by direct nucleotide sequencing of a portion of the E6 gene. Employing a third primer from this gene and *in vitro* transcription from the T7 phage RNA polymerase promoter, sufficient HPV template for nucleotide sequencing was produced. The sequencing indicated that the oral and cervical HPV16 E6 genes were, as predicted from the restriction endonuclease digestions, identical over the 120 bp amplified in the initial experiments, including the primer 1 sequence. It is perhaps significant in view of the essential role of E6 gene expression in tumours that the E6 genes from a total of nine oral and nine cervical HPV16 positive samples sequenced to date show no sequence variations (manuscript in preparation).

Unfortunately, we are still unable to determine, on the basis of either the Southern blotting or the PCR results, whether the frequently negative result with the late region primers is due to lack of these sequences, or to gross sequence divergence, which would make both annealing of high complexity probes in Southern blotting or low complexity oligonucleotide primers for the PCR unlikely. The detection of multiple low molecular weight bands in the simultaneous E6 and L1/L2 priming (Figure 4) experiments probably represents self-priming of the cellular DNA, but could equally be the result of viral genome rearrangements. The failure of these 'ladders' to hybridise to an HPV16 probe argues in favour of the former explanation, however. If the late regions were completely absent, then the oral 'sequences' would not represent a viable virus.

The resolution of these two possibilities requires cloning of the HPV16 homologous sequences from the oral cultures, which will provide the required amounts of DNA. Using the amplified sequence for the E6 region as an homologous hybridisation probe, we are now able to clone the entire HPV16-related sequence and to determine its role in oral diseases, including cancer.

The use of the PCR has overcome some of the extreme difficulties experienced in the analysis, at the molecular level, of extremely small tissue samples and heterogeneous primary cell cultures. The technique should also assist in epidemiological studies of virus incidence, without the compromises in sensitivity which are often necessary in the handling of large sample numbers.

We wish to thank Cliff Jeal and Sue Hagan for their excellent assistance with matters photographic, and Caroline Lynas for assistance with developing and running the PCR. This work was supported by the Cancer Research Campaign.

### References

BAKER, C.C., PHELPS, W.C., LINDGREN, V., BRAUN, M.J., GONDA, M.A. & HOWLEY, P.M. (1987). Structural and transcriptional analysis of human papillomavirus type sequences in cervical carcinoma cell lines. *J. Virol.*, **61**, 962.

BOS, J.L., FEARON, E.R., HAMILTON, S.R. and 4 others (1987). Prevalence of ras gene mutations in human colorectal cancers. *Nature*, **327**, 293.

CHEHAB, F.F. DOHERTY, M., CAI, S., KAN, Y.W., COOPER, S. & RUBIN, E.M. (1987). Detection of sickle cell anaemia and thalassaemias. *Nature*, **329**, 293.

CHIRGWIN, J.M., PRZBYLA, A.E., MACDONALD, R.J. & RUTTER, W.J. (1979). Isolation of biologically active ribonucleic acid from sources enriched in ribonucleases. *Biochemistry*, **18**, 5294.

CRANE, I.J., LUKER, J., STONE, A., SCULLY, C. & PRIME, S.S. (1986). Characterisation of malignant rat keratinocytes in culture following the induction of squamous cell carcinoma *in vivo*. *Carcinogenesis*, **7**, 1723.

DAVANALOO, P., ROSENBERG, A.H., DUNN, J.J. & STUDIER, F.W. (1984). Cloning and expression of the gene for bacteriophage T7 RNA polymerase. *Proc. Natl Acad. Sci. USA*, **81**, 2035.

DE VILLIERS, E.-M., WEIDAVER, H., OTTO, H. & ZUR HAUSEN, H. (1985). Papillomavirus DNA in human tongue carcinomas. *Int. J. Cancer*, **36**, 575.

HIGUCHI, R., VON BEROLDINGEN, C.H., SENSABAUGH, G.F.. & ERLICH, H.A. (1988). DNA typing from single hairs. *Nature*, **332**, 543.

HOWLEY, P.M. (1986). On human papillomaviruses. *N. Engl. J. Med.*, **315**, 1089.

LONING, T.H., IKENBERG, H., BECKER, J., GISSMAN, L., HOEPFNER, I. & ZUR HAUSEN, H. (1985). Analysis of oral papillomas, leukoplakias and invasive carcinomas for human papillomavirus type related DNA. *J. Invest., Dermatol.*, **88**, 417.

MAITLAND, N.J., COX, M.F., LYNAS, C., PRIME, S.S. & SCULLY, C. (1987*a*). Detection of human papillomavirus DNA in biopsies of human oral tissue. *Br. J. Cancer*, **56**, 245.

MAITLAND, N.J., COX, M.F., PRIME, S.S., CRANE, I.J. & SCULLY, C. (1987*b*). Nucleic acid probes in the study of latent viral disease. *J. Oral Pathol.*, **16**, 199.

MUNOZ, N., BOSCH, X. & KALDOR, J.M. (1988). Does human papillomavirus cause cervical cancer? The state of the epidemiological evidence. *Br. J. Cancer*, **57**, 1.

RHEINWALD, J.G. & GREEN, H. (1975). Serial cultivation of strains of human epidermal keratinocytes: the formation of keratinizing colonies from single cells. *Cell*, **6**, 331.

SAIKI, R.K., BUGAWAN, T.L., HORN, G.T., MULLIS, K.B. & EHRLICH, H.A. (1986). Analysis of enzymatically amplified globin and HLA-DQ DNA with allele specific probes. *Nature*, **334**, 163.

SAIKI, R.K., GELFAND, D.H., STOFFEL, S. and 5 others (1988). Primer directed enzymatic amplification of DNA with a thermostable DNA polymerase. *Science*, **239**, 487.

SCULLY, C., COX, M.F., PRIME, S.S. & MAITLAND, N.J. (1988). Papillomaviruses: the current status in relation to oral disease. *Oral Surg.*, **65**, 526.

SCULLY, C., PRIME, S.S., MAITLAND, N.J. & COX, M.F. (1987). Human papillomavirus in biopsies of human oral tissue. *Lancet*, **i**, 336.

SEEDORF, K., KRAMMER, G., DURST, M., SUHAI, S. & ROWEKAMP, W.G. (1985). Human papillomavirus type 16 DNA sequence. *Virology*, **145**, 181.

SHIBATA, D., ARNHEIM, N.J. & MARTIN, W.J. (1988). Detection of human papillomavirus in paraffin-embedded tissue using the polymerase chain reaction. *J. Exp. Med.*, **167**, 225.

SMOTKIN, D. & WETTSTEIN, F.O. (1986). Transcription of human papillomavirus type 16 early genes in a cervical cancer and a cervical cancer-derived cell line and identification of the E7 protein. *Proc. Natl Acad. Sci. USA*, **83**, 4680.

STOFLET, E.S., KOEBERL, D.D., SARKAR, G. & SOMMER, S.S. (1988). Genomic amplification with transcript sequencing. *Science*, **239**, 491.


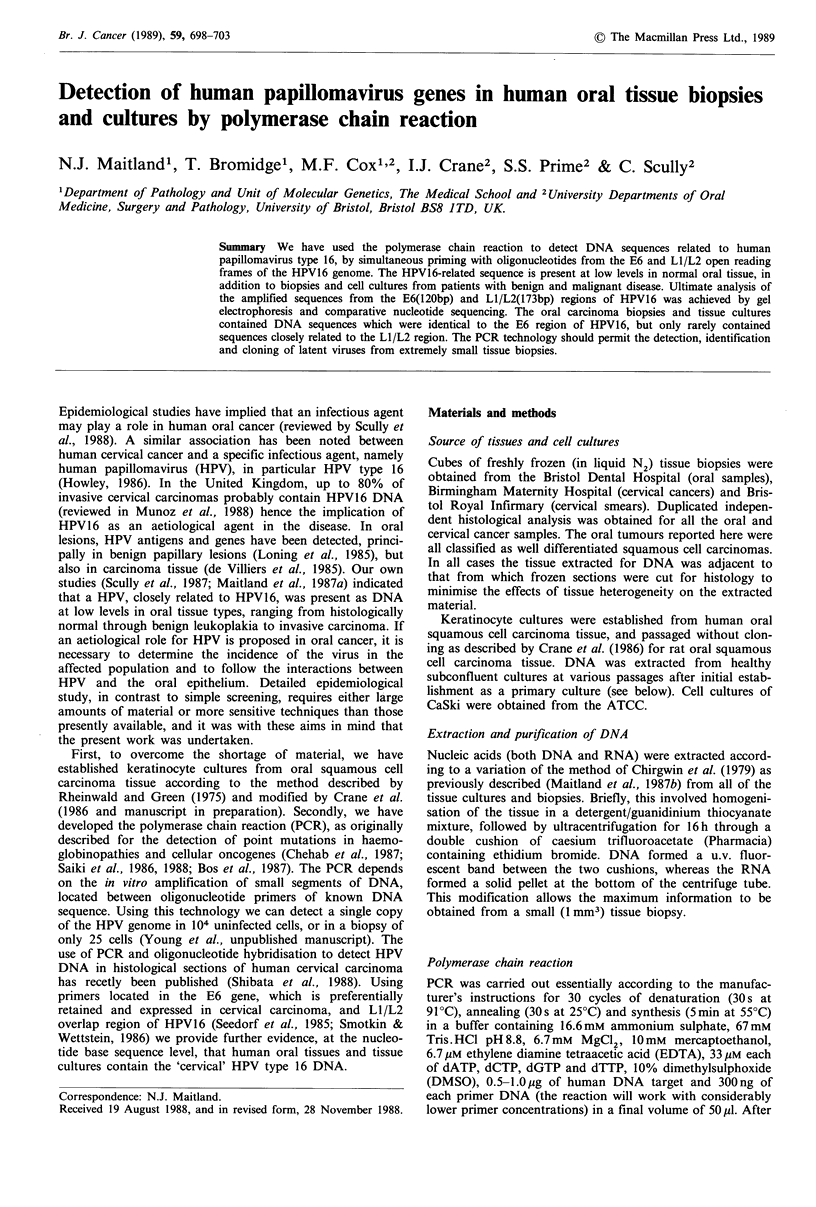

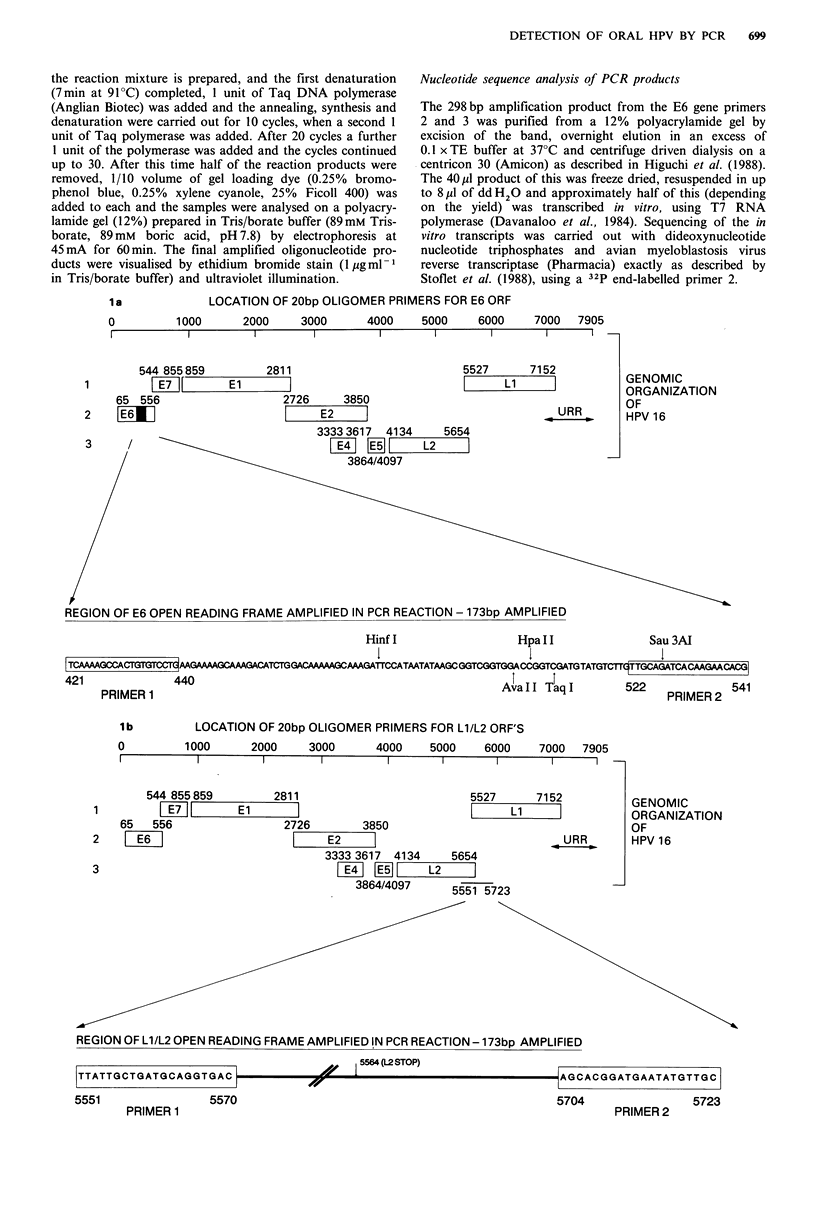

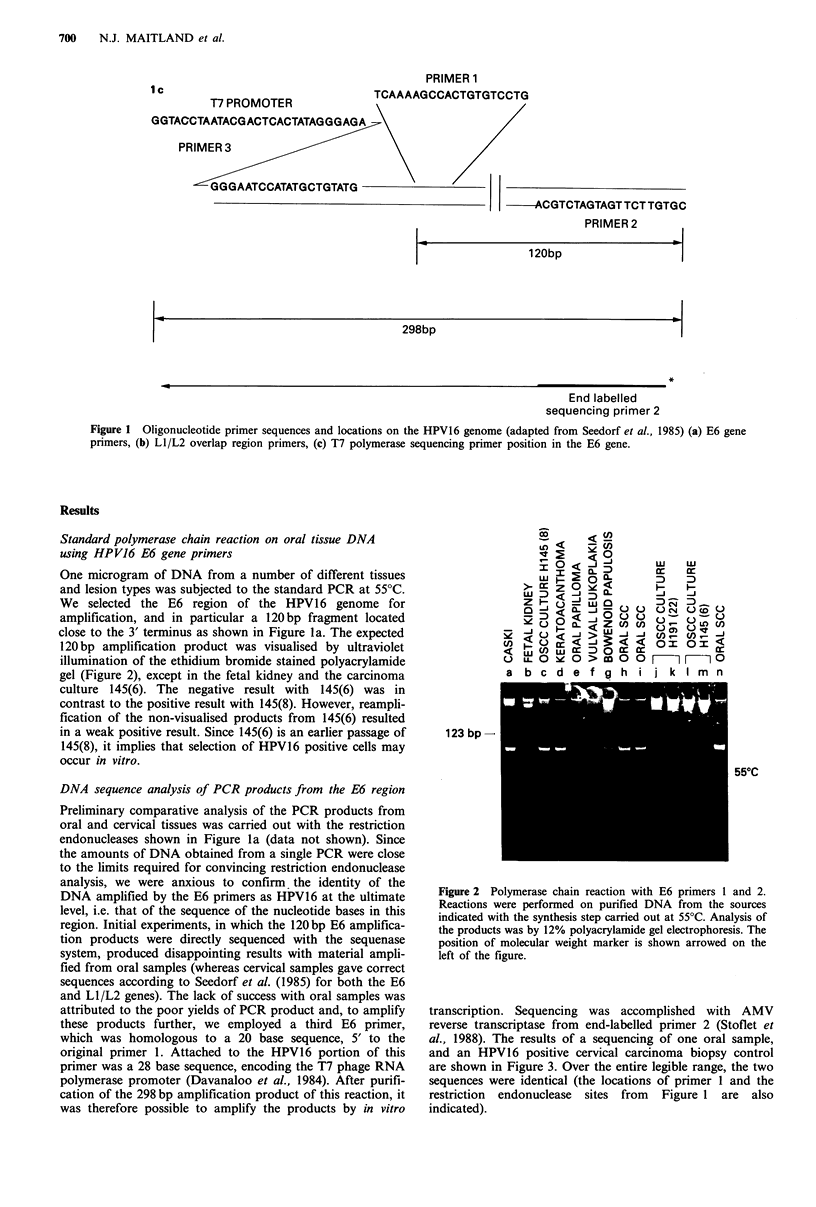

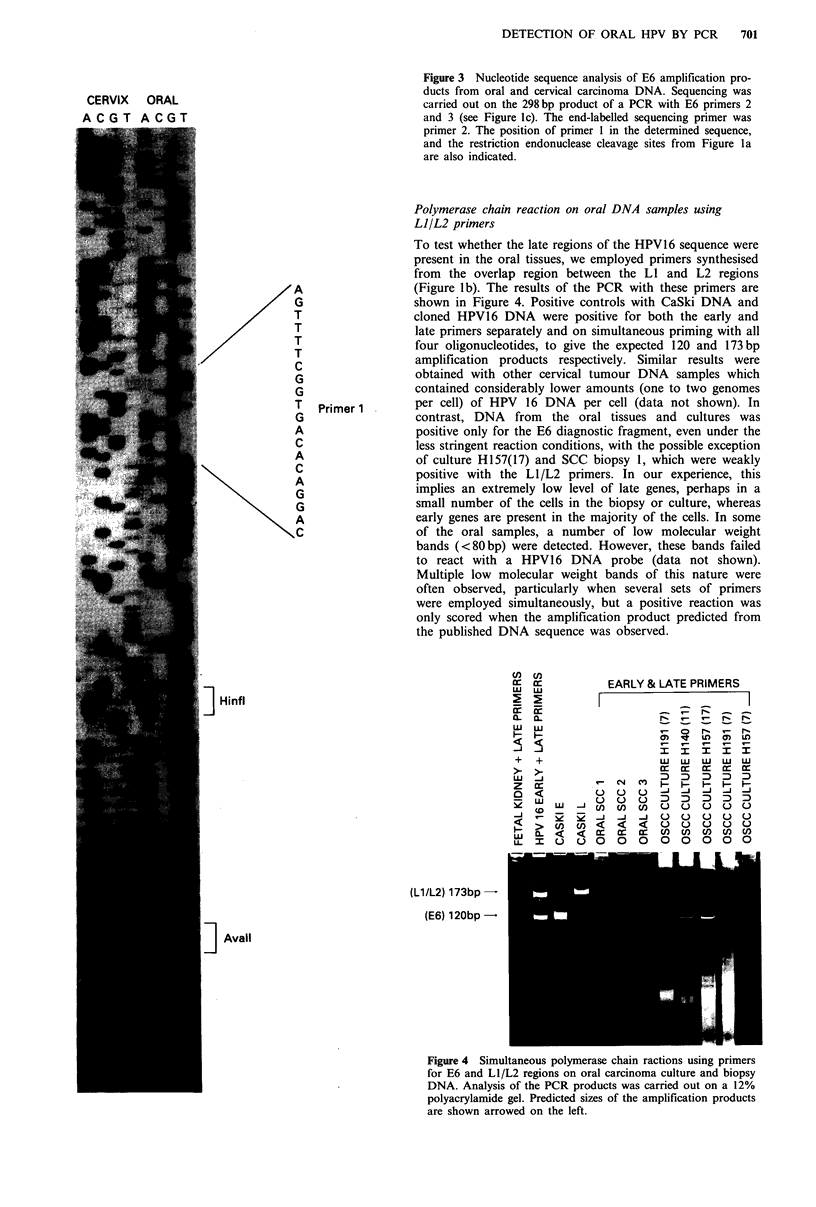

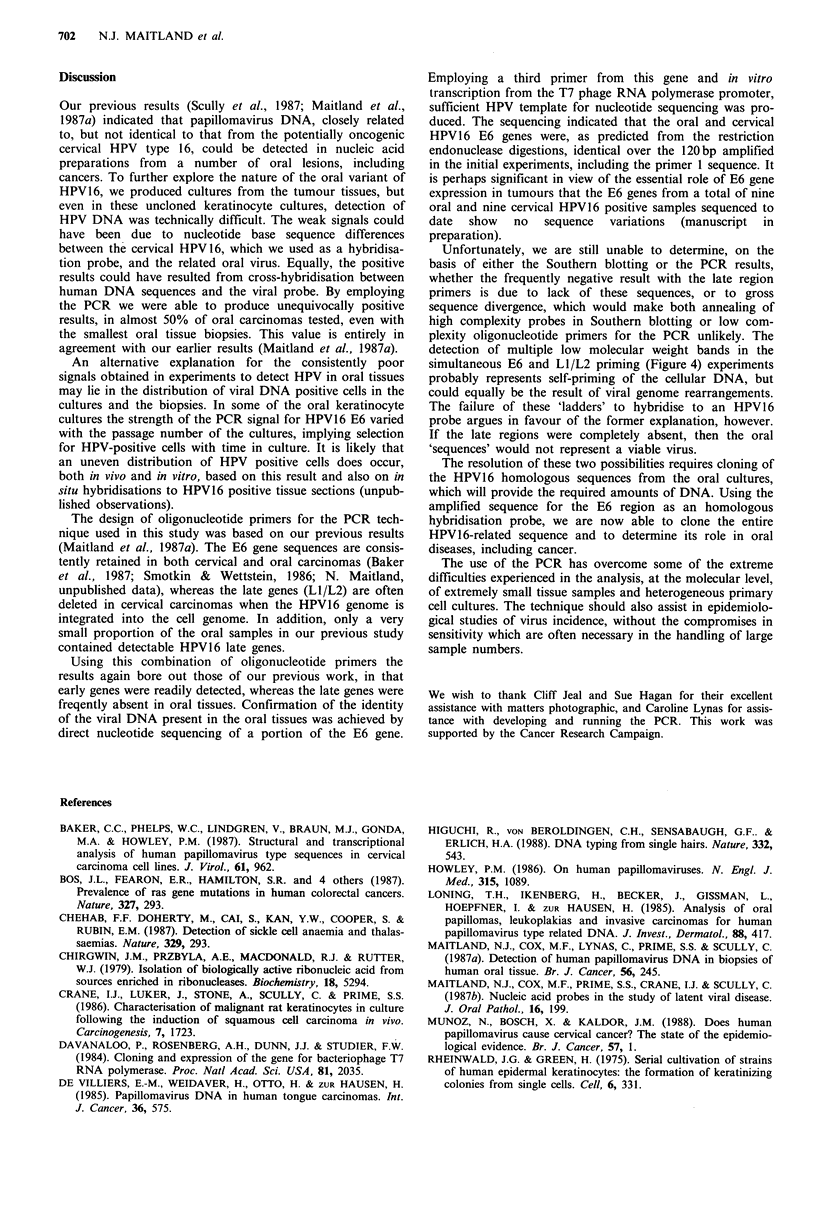

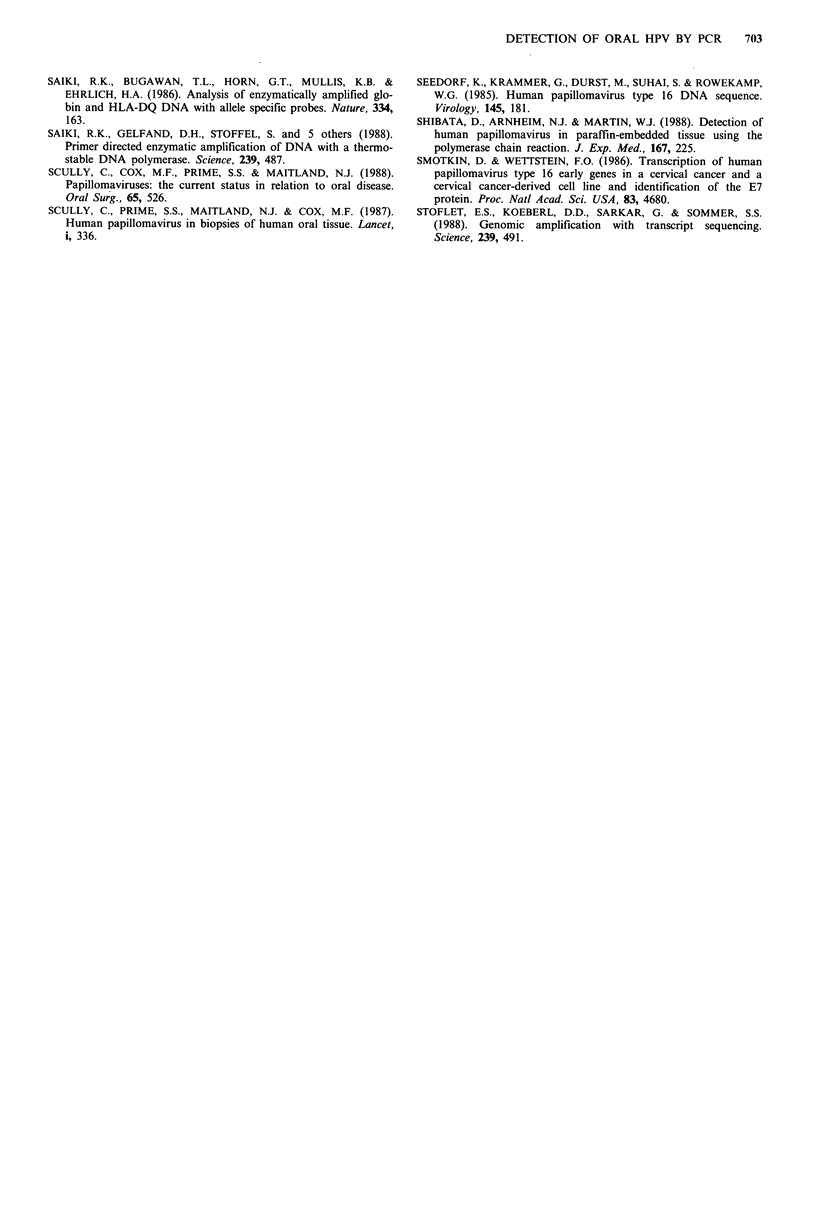


## References

[OCR_00587] Baker C. C., Phelps W. C., Lindgren V., Braun M. J., Gonda M. A., Howley P. M. (1987). Structural and transcriptional analysis of human papillomavirus type 16 sequences in cervical carcinoma cell lines.. J Virol.

[OCR_00593] Bos J. L., Fearon E. R., Hamilton S. R., Verlaan-de Vries M., van Boom J. H., van der Eb A. J., Vogelstein B. Prevalence of ras gene mutations in human colorectal cancers.. Nature.

[OCR_00600] Chehab F. F., Doherty M., Cai S. P., Kan Y. W., Cooper S., Rubin E. M. (1987). Detection of sickle cell anaemia and thalassaemias.. Nature.

[OCR_00603] Chirgwin J. M., Przybyla A. E., MacDonald R. J., Rutter W. J. (1979). Isolation of biologically active ribonucleic acid from sources enriched in ribonuclease.. Biochemistry.

[OCR_00608] Crane I. J., Luker J., Stone A., Scully C., Prime S. S. (1986). Characterization of malignant rat keratinocytes in culture following the induction of oral squamous cell carcinomas in vivo.. Carcinogenesis.

[OCR_00614] Davanloo P., Rosenberg A. H., Dunn J. J., Studier F. W. (1984). Cloning and expression of the gene for bacteriophage T7 RNA polymerase.. Proc Natl Acad Sci U S A.

[OCR_00624] Higuchi R., von Beroldingen C. H., Sensabaugh G. F., Erlich H. A. (1988). DNA typing from single hairs.. Nature.

[OCR_00629] Howley P. M. (1986). On human papillomaviruses.. N Engl J Med.

[OCR_00633] Löning T., Ikenberg H., Becker J., Gissmann L., Hoepfer I., zur Hausen H. (1985). Analysis of oral papillomas, leukoplakias, and invasive carcinomas for human papillomavirus type related DNA.. J Invest Dermatol.

[OCR_00638] Maitland N. J., Cox M. F., Lynas C., Prime S. S., Meanwell C. A., Scully C. (1987). Detection of human papillomavirus DNA in biopsies of human oral tissue.. Br J Cancer.

[OCR_00643] Maitland N. J., Cox M. F., Lynas C., Prime S., Crane I., Scully C. (1987). Nucleic acid probes in the study of latent viral disease.. J Oral Pathol.

[OCR_00648] Muñoz N., Bosch X., Kaldor J. M. (1988). Does human papillomavirus cause cervical cancer? The state of the epidemiological evidence.. Br J Cancer.

[OCR_00653] Rheinwald J. G., Green H. (1975). Serial cultivation of strains of human epidermal keratinocytes: the formation of keratinizing colonies from single cells.. Cell.

[OCR_00660] Saiki R. K., Bugawan T. L., Horn G. T., Mullis K. B., Erlich H. A. (1986). Analysis of enzymatically amplified beta-globin and HLA-DQ alpha DNA with allele-specific oligonucleotide probes.. Nature.

[OCR_00666] Saiki R. K., Gelfand D. H., Stoffel S., Scharf S. J., Higuchi R., Horn G. T., Mullis K. B., Erlich H. A. (1988). Primer-directed enzymatic amplification of DNA with a thermostable DNA polymerase.. Science.

[OCR_00671] Scully C., Cox M. F., Prime S. S., Maitland N. J. (1988). Papillomaviruses: the current status in relation to oral disease.. Oral Surg Oral Med Oral Pathol.

[OCR_00676] Scully C., Maitland N. J., Cox M. F., Prime S. S. (1987). Human papillomavirus DNA and oral mucosa.. Lancet.

[OCR_00681] Seedorf K., Krämmer G., Dürst M., Suhai S., Röwekamp W. G. (1985). Human papillomavirus type 16 DNA sequence.. Virology.

[OCR_00686] Shibata D. K., Arnheim N., Martin W. J. (1988). Detection of human papilloma virus in paraffin-embedded tissue using the polymerase chain reaction.. J Exp Med.

[OCR_00691] Smotkin D., Wettstein F. O. (1986). Transcription of human papillomavirus type 16 early genes in a cervical cancer and a cancer-derived cell line and identification of the E7 protein.. Proc Natl Acad Sci U S A.

[OCR_00697] Stoflet E. S., Koeberl D. D., Sarkar G., Sommer S. S. (1988). Genomic amplification with transcript sequencing.. Science.

[OCR_00619] de Villiers E. M., Weidauer H., Otto H., zur Hausen H. (1985). Papillomavirus DNA in human tongue carcinomas.. Int J Cancer.

